# A noninvasive artificial neural network model to predict IgA nephropathy risk in Chinese population

**DOI:** 10.1038/s41598-022-11964-5

**Published:** 2022-05-18

**Authors:** Jie Hou, Shaojie Fu, Xueyao Wang, Juan Liu, Zhonggao Xu

**Affiliations:** Department of Nephrology, The First Hospital of Jilin University, Changchun, 130021 Jilin China

**Keywords:** Mathematics and computing, Nephrology, Kidney diseases

## Abstract

Renal biopsy is the gold standard for Immunoglobulin A nephropathy (IgAN) but poses several problems. Thus, we aimed to establish a noninvasive model for predicting the risk probability of IgAN by analyzing routine and serological parameters. A total of 519 biopsy-diagnosed IgAN and 211 non-IgAN patients were recruited retrospectively. Artificial neural networks and logistic modeling were used. The receiver operating characteristic (ROC) curve and performance characteristics were determined to compare the diagnostic value between the two models. The training and validation sets did not differ significantly in terms of any variables. There were 19 significantly different parameters between the IgAN and non-IgAN groups. After multivariable logistic regression analysis, age, serum albumin, serum IgA, serum immunoglobulin G, estimated glomerular filtration rate, serum IgA/C3 ratio, and hematuria were found to be independently associated with the presence of IgAN. A backpropagation network model based on the above parameters was constructed and applied to the validation cohorts, revealing a sensitivity of 82.68% and a specificity of 84.78%. The area under the ROC curve for this model was higher than that for logistic regression model (0.881 vs. 0.839). The artificial neural network model based on routine markers can be a valuable noninvasive tool for predicting IgAN in screening practice.

## Introduction

Immunoglobulin A nephropathy (IgAN) is currently the most common primary glomerular disease worldwide and accounts for more than 40% of primary glomerular diseases in China^1,2^. IgAN has diverse clinical manifestations and complicated pathogenesis. The most common clinical presentations are asymptomatic hematuria, proteinuria, and renal insufficiency. Epidemiological studies have found that patients with IgAN can progress to end-stage renal disease (ESRD) within 10–20 years after the first detection^3^. The diagnosis and treatment of IgAN have become a prominent problem in the field of nephrology, and early diagnosis has great clinical significance for delaying the progression of the disease.

Renal biopsy is the gold standard for diagnosing IgAN. However, it is an invasive operation involving complications such as bleeding and infection^4^. Due to the lack of advanced equipment in basic hospitals, this procedure is difficult to perform in some less-developed areas, making the best treatment inaccessible to many patients. There is an urgent need for a simple and noninvasive diagnostic model for IgAN. In the era of big data, computational models known as artificial neural networks (ANNs) have nonlinear functions, which can deal with the complex relationships between input and output. The ANN model can analyze the interactions among medical risk factors more clearly than traditional statistical approaches by learning from examples^5,6^. It has been widely used to predict, diagnose, and classify various diseases in the life sciences^7–9^. The backpropagation ANN (BP-ANN) algorithm, one of the most popular neural network models, is a multilayer feed-forward network that depends on the error backpropagation algorithm^10^. Presently, the risk prediction of IgAN prognosis has been widely studied both locally and abroad^11,12^. However, studies concerning the early risk prediction model of IgAN are still limited. As a result, noninvasive predictive models using BP-ANN and traditional logistic regression are expected to be constructed and compared.

The present study aimed to retrospectively analyze the biological parameters closely related to the presence of IgAN and to screen out optimized biological parameters. The noninvasive anticipated model can not only be used for early warning of IgAN, but also help improve disease prognosis.

## Methods

### Study subjects

Patients first diagnosed with primary glomerular diseases via renal biopsy at the First Hospital of Jilin University between November 1, 2010, and November 1, 2020, were selected as the research subjects and were divided into two groups: the IgAN group and the non-IgAN group (those diagnosed with other primary glomerular diseases). The inclusion criteria were as follows: (1) patients who consented to undergo a renal biopsy during their hospital admission, (2) those who had complete clinical information, and (3) those aged above 18 years. The exclusion criteria were as follows: (1) patients who received corticosteroids or immunosuppression treatment before their condition was diagnosed; (2) those with a history secondary kidney disease; (3) those aged under 18 years; (4) pregnant women; and (5) patients diagnosed with an infection, tumor, or autoimmune disease. Based on the above criteria, 730 cases were finally enrolled (212 patients with IgAN and 518 patients without IgAN), and they were randomly divided into a training cohort (n = 511) and a validation cohort (n = 219) in a 7:3 ratio. The training cohort was used to build the prediction models and the validation cohort was used to test the prediction effect of the models.

This study was approved by the ethics committee of the First Hospital of Jilin University, Changchun, China (2021–036).

### Clinical parameters

Clinical information such as age, sex, blood pressure, laboratory tests (including blood biochemistry, immunology, and urinalysis) before renal biopsy, and renal pathological data were collected. Then, the serum IgA/C3 ratio was calculated for each patient.

### Algorithm of BP-ANN

We explored the relationship between risk factors and IgAN using the BP-ANN model. The BP-ANN was composed of three layers: the input layer, hidden layer, and output layer. The input layer of the ANN consisted of the variables showing statistical significance in the logistic regression analysis. The output layer referred to one neuron representing the presence of IgAN (valued as end = 1 for IgAN, and end = 0 for non-IgAN). The entire group was divided into a training group (70%) and a validation group (30%) using a random number generator. Back propagation of the error was used to dynamically adjust the network weights until the error was satisfied.

### Statistical analysis

Statistical analysis was performed using SPSS version 19.0. Normally distributed data were expressed as x ± s (mean ± SD) and compared using the unpaired Student’s t-test. The non-normally distributed data were expressed as medians with their corresponding interquartile ranges and compared using the Mann–Whitney U-test. Categorical variables were expressed as proportions (percentages) and compared using Chi-square tests. A value of *P* < 0.05 was considered to indicate a statistical difference. Statistically significant indicators from the univariate analysis were used as independent variables in the logistic regression model. Receiver operating characteristic (ROC) curves were then plotted, and the area under the curve (AUC) was calculated. The ANN models were developed using MATLAB 7.4.0. The predictive level of the model was evaluated based on the AUC, sensitivity, and specificity values.

### Ethics approval

This study was approved by the ethics committee of the First Hospital of Jilin University, Changchun, China (2021-036).

### Consent to participate

Written informed consent was provided from all participants.

### Consent for publication

Consent for publication can be obtained from participants.

### Statement of methods

All methods were carried out in accordance with relevant guidelines and regulations.

## Results

### Clinical characteristics

**Figure 1:**
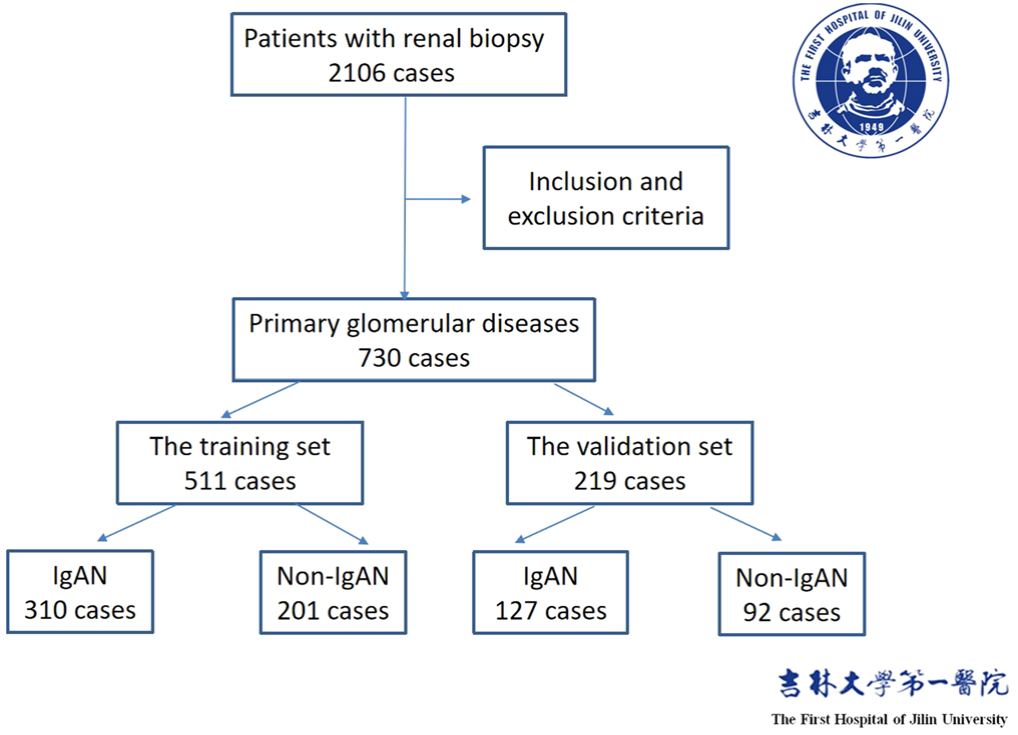
The flow diagram of subjects screening and grouping.

A total of 730 patients with a primary glomerular diseases were enrolled in this study. The pathological types of the non-IgAN cases included membranous nephropathy, mesangial capillary glomerulonephritis, focal segmental glomerulosclerosis, and minimal change disease. The flow diagram of subjects screening and grouping is shown Fig. 1. The training cohort consisted of 511 patients (310 with IgAN and 201 with non-IgAN), of which 45.5% were male, with an average age of 39 years (range, 29.0–51.8 years). The validation set consisted of 219 patients (127 with IgAN and 92 with non-IgAN), of which 47% were male, with an average age of 40 years (range, 30.0–52.0 years). As shown in Table 1, there were no statistical differences in any clinical characteristics between the training and validation cohorts.

**Table 1:** Clinical characteristics of the training and validation groups.

	Training groups n = 511	Validation group n = 219	*P* value
Age	39 (29.0–51.8)	40 (30.0–52.0)	0.392
Female	279 (54.5%)	116 (53%)	0.766
Hypertention	175 (34.2%)	78 (35.6%)	0.772
Hematuria	280 (54.7%)	125 (57.3%)	0.563
24-h urine protein (g)	2.73 (1.38–5.48)	2.80 (1.20–6.03)	0.857
Serum albumin (g/L)	30.1 (22.40–36.40)	30 (22.00–36.40)	0.981
Total cholesterol (mmol/L)	5.57 (4.36–7.44)	5.2 (4.12–7.36)	0.130
Triglycerides (mmol/L)	1.74 (1.25–2.62)	1.6 (1.12–2.60)	0.289
HDL cholesterol (mmol/L)	1.32 (1.06–1.67)	1.39 (1.09–1.73)	0.282
LDL cholesterol (mmol/L)	3.25 (2.36–4.38)	3.12 (2.25–4.40)	0.857
Serum IgA (g/L)	2.76 (1.99–3.73)	2.85 (2.07–4.02)	0.301
Serum IgG (g/L)	7.36 (4.05–10.00)	7.61 (3.81–10.70)	0.297
Serum IgM (g/L)	1.01 (0.75–1.40)	0.94 (0.69–1.40)	0.079
Complement C3 (g/L)	1.15 (1.00–1.34)	1.15 (0.97–1.31)	0.473
Serum IgA/C3 ratio	2.11 (1.54–3.06)	2.40 (1.72–3.11)	0.051
Uric acid (mmol/L)	377 (308–465)	380 (317–438)	0.310
Creatinine (µmol/L)	83.5 (65.0–114.8)	82.6 (64.85–119.8)	0.862
Urea nitrogen (mmol/L)	6.00 (4.80–8.03)	6.08 (4.80–7.86)	0.835
eGFR (mL/min/1.73 m^2^)	108.5 (69.0–129.8)	100.4 (43.4–138.0)	0.410
Hemoglobin (g/L)	134.00 (119.0–147.0)	134.50 (120.0–152.3)	0.215

IgA: immunoglobulin A; IgG: immunoglobulin G; IgM: immunoglobulin M; eGFR: estimated glomerular filtration rate.

As presented in Table 2, compared with the non-IgAN group patients in the training cohort, the IgAN group patients were significantly younger on average, had a higher incidence of hematuria and hypertension, and had higher levels of serum albumin, urea nitrogen, creatinine, uric acid, IgA, IgG, and IgA/C3 ratios (*P* < 0.01). The IgAN group patients also had significantly lower levels of serological IgM, complement C3, total cholesterol, triglycerides, high-density lipoprotein (HDL) cholesterol, low-density lipoprotein(LDL) cholesterol, and hemoglobin, as well as a significantly lower estimated glomerular filtration rate (eGFR) and 24-h urine protein (*P* < 0.01).

**Table 2:** Differences in the clinical parameters between IgAN and non-IgAN in the training set.

	IgAN n = 310	Non-IgAN n = 201	*P* value
Age	37.92 (28.0–46.3)	47.0 (31.75–56.7)	< 0.001
Female	179 (57.6%)	100 (49.8%)	0.101
Hypertention	128 (41.2%)	47 (23.14%)	< 0.001
Hematuria	194 (62.4%)	86 (42.8%)	< 0.001
24-h urine protein (g)	2.10 (1.13–3.78)	4.39 (2.10–10.07)	< 0.001
Serum albumin (g/L)	34.10 (29.10 − 37.90)	22.20 (16.00–28.37)	< 0.001
Total cholesterol (mmol/L)	4.90 (4.19–5.99)	7.30 (5.34–9.79)	< 0.001
Triglycerides (mmol/L)	1.63 (1.18–2.50)	1.89 (1.35–2.94)	0.002
HDL cholesterol (mmol/L	1.20 (0.99–1.51)	1.56 (1.21–1.94)	< 0.001
LDL cholesterol (mmol/L)	2.81 (2.29–3.60)	4.25 (3.02–6.59)	< 0.001
Serum IgA (g/L)	3.20 (2.49–4.15)	2.12 (1.53–2.82)	< 0.001
Serum IgG (g/L)	9.16 (6.44–11.30)	4.50 (2.96–6.73)	< 0.001
Serum IgM (g/L)	0.94 (0.71–1.29)	1.15 (1.53–2.82)	< 0.001
Complement C3 (g/L)	1.08 (0.97–1.27)	1.24 (1.08–1.44)	< 0.001
Serum IgA/C3 ratio	2.71 (2.04–3.48)	1.64 (1.15–2.16)	< 0.001
Uric acid (mmol/L)	448 (326–480)	297 (293–420)	< 0.001
Creatinine (µmol/L)	94.30(73.70–138.03)	68.55(55.68–86.03)	< 0.001
Urea nitrogen (mmol/L)	6.20(4.92–8.30)	5.60(4.32–7.83)	0.007
eGFR (mL/min/1.73 m^2^)	79.38 (52.31–99.40 )	103.61 (82.35–114.87)	< 0.001
Hemoglobin (g/L)	130.75 ± 20.96	135.45 ± 6 22.75	0.011

IgA: immunoglobulin A; IgG: immunoglobulin G; IgM: immunoglobulin M; eGFR: estimated glomerular filtration rate.

### Univariate and multivariate logistic regression

**Figure 2:**
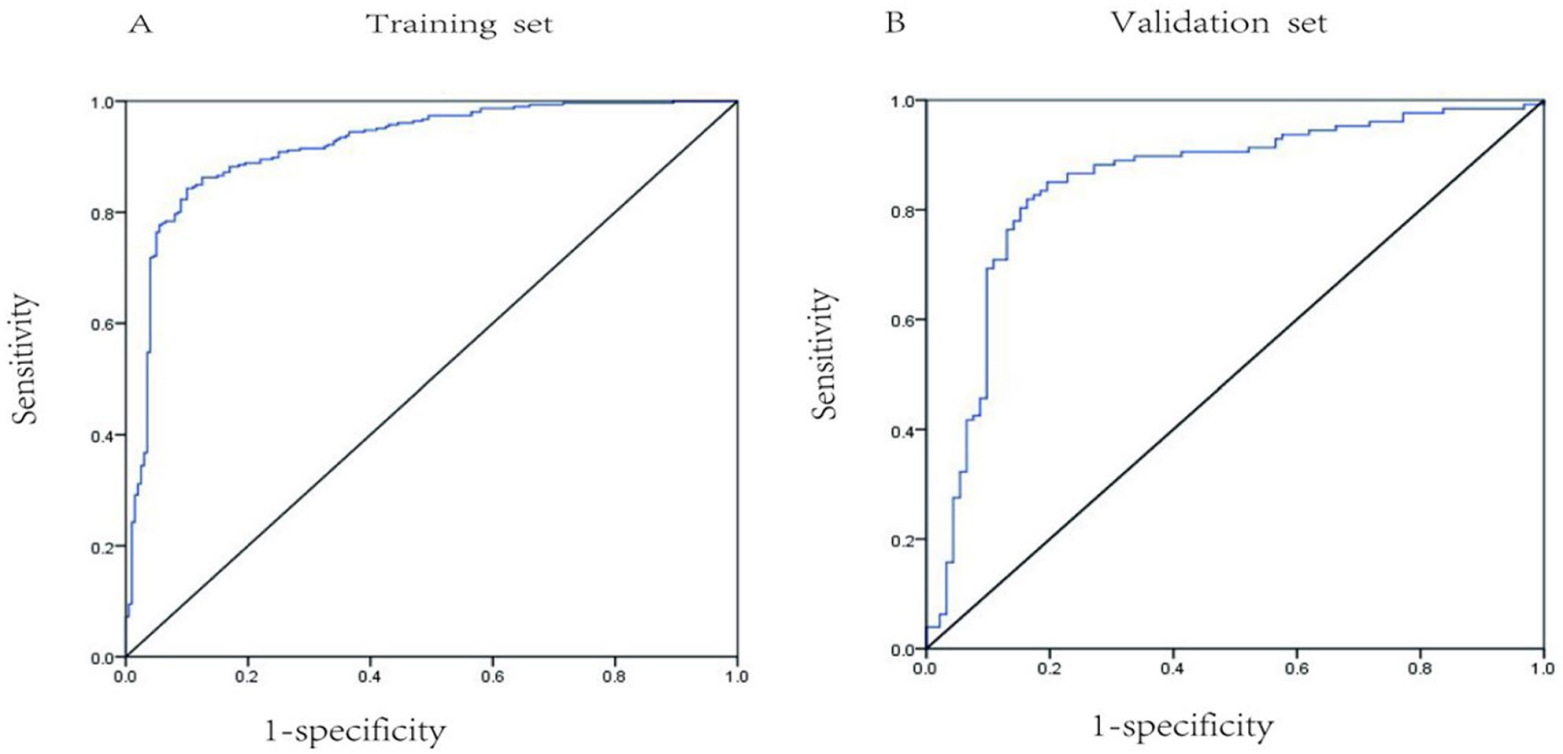
ROC curve of logistic regression modeling for predicting IgAN. (**A**) Area under the ROC curves were 0.92 in training set. (**B**) Area under the ROC curves were 0.839 in validation set.

Univariate analysis of the training cohort revealed that the following 19 items were significantly related to IgAN (all *P* < 0.05): age, hypertension, hematuria, eGFR, 24-h urine protein and serum albumin, urea nitrogen, creatinine, uric acid, IgA, IgG, IgM, complement C3, IgA/C3 ratio, total cholesterol, triglycerides, HDL cholesterol, LDL cholesterol, and hemoglobin. After considering the covariate collinearity among the aforementioned factors, urea nitrogen, creatinine, and hemoglobin were excluded. The other 16 significant variables were included in the multivariable logistic analysis. Our results showed that age, serum IgA/C3 ratio, serum albumin, serum IgA, serum IgG, eGFR, and hematuria were independent risk indicators of the occurrence of IgAN (Table 3). The abovementioned seven factors were selected as parameters to establish a regression equation for the diagnosis of IgAN, expressed as P = exp(− 0.808 − 0.051 × age + 0.099 × albumin + 0.766 × hematuria + 0.349 × IgA + 0.134 × IgG + 0.709 × IgA/C3 ratio − 0.028 × eGFR)/[1 + exp(0.808 − 0.051 × age + 0.099 × albumin + 0.766 × hematuria + 0.349 × IgA + 0.134 × IgG + 0.709 × IgA/C3 ratio − 0.028 × eGFR)]. The ROC curve was plotted, and the AUC, sensitivity, and specificity were estimated to be 0.92, 84.1%, and 91.4%, respectively (Fig. 2A). When applied to the test dataset, the logistic regression model showed an AUC of 0.839, a sensitivity of 81.9%, and a specificity of 83.7% (Fig. 2B).

**Table 3:** Multivariate logistic regression analysis for IgAN.

	B	*P*	OR	95% C.I. for EXP(B)	
				Lower	Upper
Age	− 0.051	< 0.001	0.947	0.928	0.967
Albumin	0.099	< 0.001	1.102	1.060	1.145
Hematuria	0.766	0.020	1.947	1.109	3.418
Serum IgA	0.349	< 0.001	1.472	1.192	1.817
Serum IgG	0.134	0.014	1.145	1.027	1.275
Serum IgA/C3 Ratio	0.709	< 0.001	2.039	1.401	2.967
eGFR	− 0.028	< 0.001	0.972	0.962	0.981
Constant	− 1.808	0.033	0.164

### BP-ANN model prediction of IgAN

**Figure 3:**
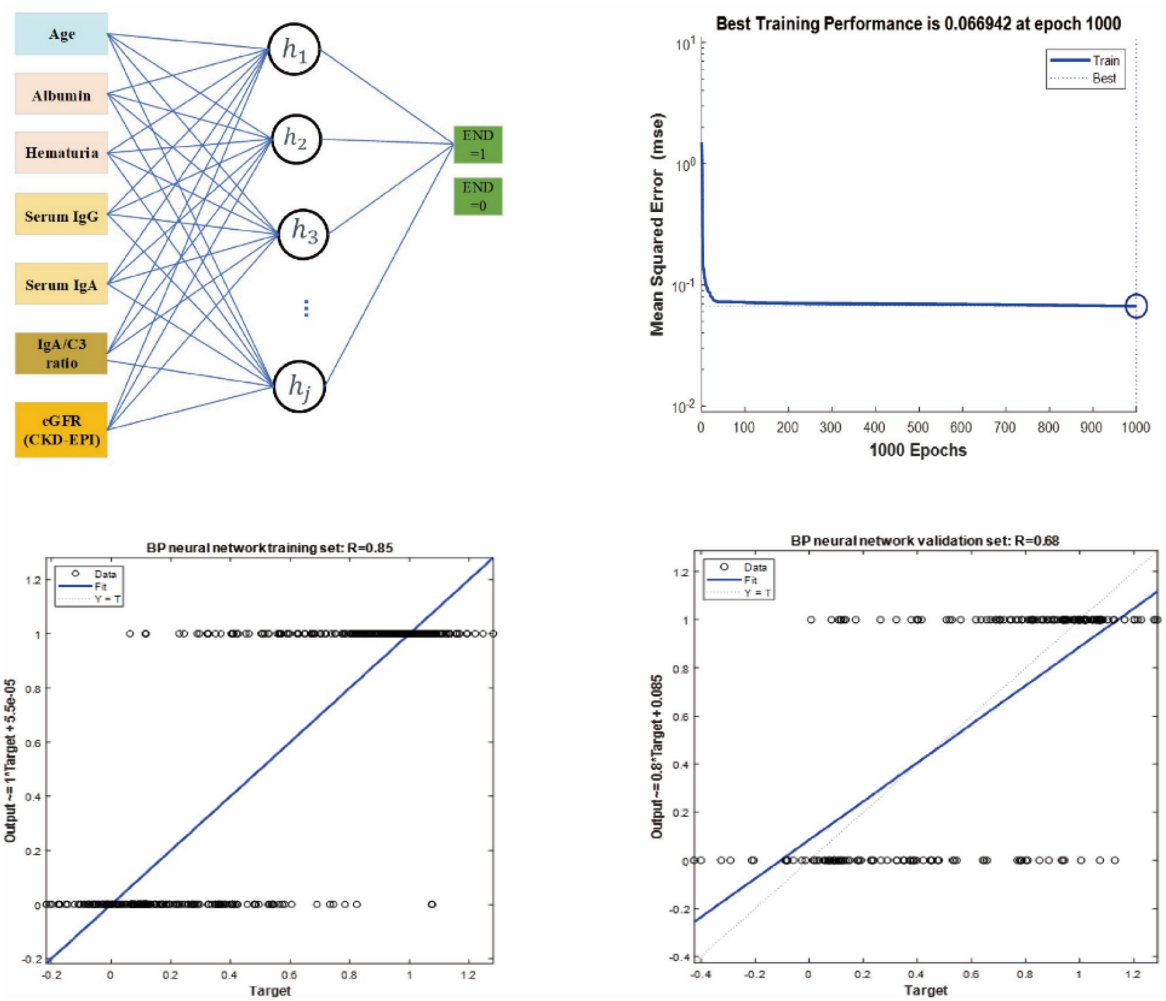
The structure of the artificial neural networks model and BP-ANN training process.

**Figure 4:**
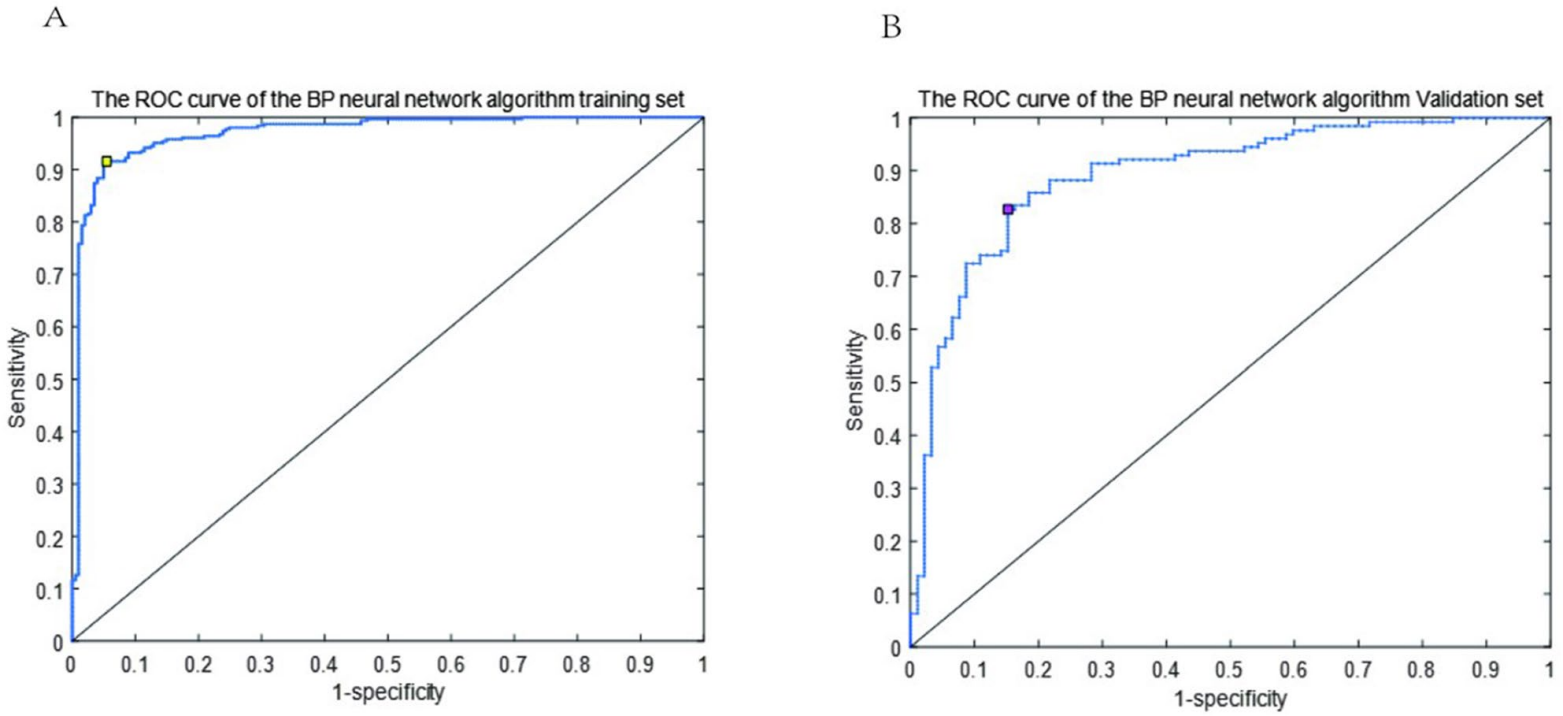
ROC curve of BP-ANN for predicting IgAN. (**A**) Area under the ROC curves were 0.965 in training set. (**B**) Area under the ROC curves were 0.881 in validation set.

A BP-ANN model was constructed using the training data. Based on the multivariable logistic regression results, seven significant factors were chosen as independent variables. The structure of BP-ANN model and network training process were shown in Fig. 3. The ROC curve was then obtained (Fig. 4A), and the BP-ANN model was found to provide a good predictive performance, with an AUC, sensitivity, and specificity of 0.965, 84.78%, and 94.53%, respectively. The predictive efficacy of the model was further evaluated using the validation set. In the validation cohort, the AUC, sensitivity, and specificity of the model were 0.881, 82.68%, and 84.78%, respectively (Fig. 4B).

### Comparison of the BP-ANN and logistic regression models

The evaluation indexes of the BP-ANN and the logistic regression models were compared. AUC values were obtained from the logistic regression and BP-ANN models using the validation set for IgAN prediction. The AUC value of the BP-ANN model was 0.881, which was higher than that of the logistic regression model, indicating the superior performance of the constructed neural network in IgAN prediction.

## Discussion

The clinical manifestations of IgAN vary from asymptomatic hematuria or proteinuria in the early stages to rapid-onset ESRD in the late stages. IgAN is generally immune-mediated by increased aberrantly glycosylated IgA1 and subsequent complement C3 deposits in the glomerular mesangium^13^. Although renal biopsy is the gold standard for diagnosing IgAN, its clinical application is limited in less-developed areas in China and by some patients’ insufficient awareness of its necessity. Galactose-deficient IgA1, a peptide mass fingerprint, has been reported as new specific indicators for the diagnosis of IgAN^14^. However, their detection costs are expensive, and technology requirements of the operation are so high that they are difficult to apply in clinical practice. Therefore, exploring the clinical and laboratory indicators related to IgAN and constructing noninvasive prediction models to screen patients with high risk are of great significance. Through a retrospective cohort study, we identified the risk factors related to IgAN, built the diagnostic models, and evaluated the predictive ability of different modeling algorithms.

In this study, compared with non-IgAN patients, IgAN was found to usually occur in young and middle-aged people, who were more likely to have hematuria, proteinuria, and hypertension. Most patients had elevated serum immunoglobulins (especially IgA), decreased complement C3, and renal injury, which are well-known features of IgAN^15^. Most scholars now agree that the serum IgA/C3 ratio is more valuable than serum IgA and C3 for the diagnosis and monitoring of IgAN^16–18^.Therefore, the serum IgA/C3 ratio was included in this study. In addition, serum IgG levels in the IgAN group were significantly higher than those in the non-IgAN group, which is consistent with the findings of previous literature^19^. In the training dataset, 70% of patients in the non-IgAN group had nephrotic syndrome, while only 22% of patients in the IgAN group had this syndrome. Because of the low proportion of nephrotic syndrome patients with IgAN, it is speculated that indicators related to nephrotic syndrome may be helpful for the differential diagnosis of IgAN and non-IgAN^20^, which also explains the high lipid and proteinuria levels and low serum albumin levels in the non-IgAN group. Logistic regression analysis was used to control for confounding factors, and seven variables, such as age, serum IgA/C3 ratio, serum albumin, serum IgA, serum IgG, eGFR, and the presence of hematuria, were found to be independent predictors of IgAN. Of these, the finding that serum IgA/C3 ratio can help diagnose IgAN is in line with previous related studies^21,22^. Originally, Maeda reported that the serum IgA/C3 ratio, combined with microscopic hematuria and/or proteinuria and high serum IgA levels, can be used to distinguish IgAN from other primary renal diseases^23^. In 2012, Gao’s team used logistic regression analysis for the differential diagnosis of IgAN and non-IgAN, by incorporating three factors: serum IgA, fibrinogen, and clinical presentation with an AUC of 0.838^24^. However, the sample size of their study was small, and therefore, the reliability of the results obtained need further verification. Later, Han QX incorporated age, serum IgA, total cholesterol, D-dimer, and fibrinogen into a logistic regression model for the noninvasive differential diagnosis of IgAN. However, the model was not validated, and therefore, its accuracy remains unverified^25^. In contrast, our study enrolled a large number of patients for the combined diagnosis of IgAN through the multiple predictors mentioned above, with an AUC of 0.92, sensitivity of 84.1%, and specificity of 91.4%. We tested the model on the validation set and obtained AUC, sensitivity, and specificity of 0.839 (more than 0.7), 81.9%, and 83.7% (more than 70%), respectively. Our results indicated that the multifactor-based logistic regression model can effectively predict the risk of IgAN.

However, logistic regression models cannot handle complex nonlinear relationships between inputs and outputs, nor can they detect all possible interactions between predictors. A logistic regression model can only work if the states of all the variables are known, which is often difficult to achieve in clinical practice. In contrast, ANNs have strong nonlinear mapping capability and can handle the complex intrinsic relationships between the missing data and variables. Furthermore, ANN models have been successfully used for prediction and classification in different areas, including informatics and medicine^26–28^. In this study, an ANN model for the early screening of IgAN was constructed and validated for the first time based on routine and serum markers. A ROC curve was used to assess the efficacy of the model in predicting the risk of IgAN. The AUC of the validation cohort was very similar to that of the training set, and both were significantly higher than those of the logistic regression model, indicating that the ANN model has better diagnostic performance in differentiating IgAN from non-IgAN. The results showed that the ANN model is more suitable for predicting the risk of IgAN than non-IgAN. Thus, it can be concluded that the ANN model has better clinical usability, as an auxiliary tool for early discovery and timely treatment.

This study developed and validated a predictive model for screening the high risk of IgAN with the following advantages: (1) all patients enrolled had primary glomerular diseases confirmed by renal biopsy; (2) combining serum IgA/C3 ratio with age, serum albumin, total cholesterol, and hematuria to establish a predictive model reduced the limitations of using only the serum IgA/C3 ratio as the differential indicator; (3) with the same modeling variables, a simple, safe, and accurate predictive model for IgAN was developed that has good prospects for clinical application.

However, we have to point out some limitations: (1) there could have been selection bias and information bias owing to the retrospective nature of study design; (2) the small size of the cohort could have influenced the model performance to some degree. Our research objective would be better addressed using a larger validation cohort in a multicenter study; and (3) the model can not determine the grade of IgAN. which has a certain impact on the diagnosis and treatment. (4) The model has not been validated in an external independent cohort.

In conclusion, the established multifactor diagnostic model could effectively distinguish IgAN patients from non-IgAN patients with good specificity. The ANN noninvasive diagnostic model can predict IgAN better than logistic regression and may have good clinical applicability. This model can be helpful for early detection of high-risk IgAN patients especially in less-developed regions.

## Data Availability

The datasets used and analysed during the current study are available from the corresponding author on reasonable request.
